# Bis[1,2-bis­(4-chloro­phen­yl)­ethyl­ene-1,2-dithiol­ato(1–)]nickel(II)

**DOI:** 10.1107/S2414314622001481

**Published:** 2022-02-17

**Authors:** Sydney Koehne, Bailey Mirmelli, Joel T. Mague, James P. Donahue

**Affiliations:** a Department of Chemistry, Tulane University, 6400 Freret Street, New Orleans, Louisiana 70118-5698, USA; Dublin City University, Ireland

**Keywords:** crystal structure, nickel bis­(di­thiol­ene), 4-chloro­phenyl-substituted di­thiol­ene

## Abstract

The title compound, [Ni(S_2_C_2_(C_6_H_4_-*p*-Cl)_2_)_2_] or [Ni(C_14_H_8_Cl_2_S_2_)_2_], crystallizes in the triclinic space group *P*  as pairs of mol­ecules disposed about an inversion center at the *bc* face of the cell. Close inter­molecular C—H⋯S (2.884 Å) and C—H⋯Ni (3.032 Å) contacts that are less than the sum of the van der Waals radii appear to induce slight bowing of the mol­ecular planes toward one another. The angles at which the four *p-*ClC_6_H_4_- rings join the NiS_2_C_2_ chelate rings [39.37 (9)– 53.41 (6)°] are similarly influenced by these inter­molecular contacts. In the larger packing arrangement, sheets of mol­ecules extend in the direction of the *ac* face diagonal.

## Abstract 2

Bis[1,2-bis­(4-chloro­phen­yl)­ethyl­ene-1,2-dithiol­ato(1−)]nickel(II) crystallizes as pairs of mol­ecules related by an inversion center with close inter­molecular C—H⋯S and C—H⋯Ni contacts.

## Graphical abstract

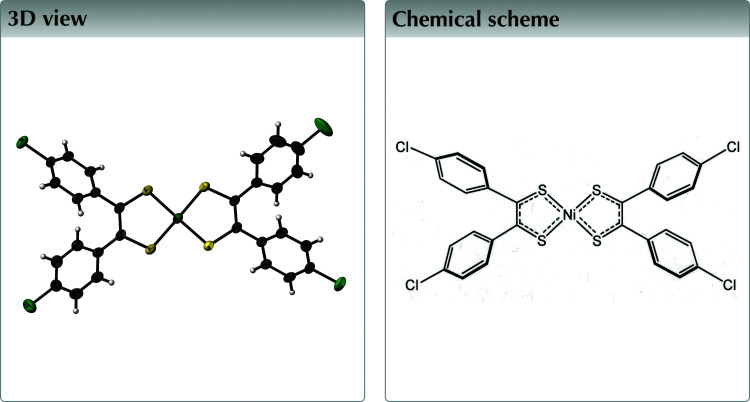

## Structure description

As seen from a survey of the Cambridge Structural Database, nickel has enjoyed the most extensive development of its coordination chemistry with di­thiol­ene ligands that bear aryl substituents. One reason for the attention given to these nickel complexes is the application they have found as reversibly bleachable Q-switching dyes for near infrared lasers (Mueller-Westerhoff *et al.*, 1991 ▸). Their photochemical, thermal, and chemical stability, in conjunction with the relative ease with which they are synthesized, has made such nickel bis­(di­thiol­ene) complexes impactful enough that a variety are now sold commercially. Charge-neutral, aryl-substituted nickel di­thiol­ene complexes, [(*R*_2_C_2_S_2_)_2_Ni], that have been structurally characterized include the complexes where *R* = Ph (Megnamisi-Belombe & Nuber, 1989 ▸; Kuramoto & Asao, 1990 ▸), *p*-CH_3_C_6_H_4_– (Miao *et al.*, 2011 ▸), *p*-CH_3_OC_6_H_4_– (Arumugam *et al.*, 2007 ▸), *p*-^*n*^BuOC_6_H_4_– (Perochon *et al.*, 2009 ▸), *p*-CH_3_(CH_2_)_11_C_6_H_4_– (Perochon *et al.*, 2009 ▸), and 3,5-(CH_3_O)_2_-4-^*n*^BuOC_6_H_2_– (Nakazumi *et al.*, 1992 ▸).

**Figure 1:**
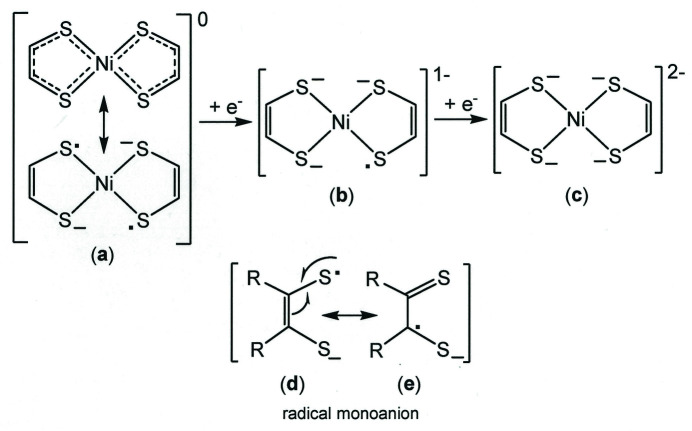
(**a**)–(**c**) Di­thiol­ene-based electron-transfer reactions within nickel(II) bis­(di­thiol­ene) complexes whereby the ligands are transformed from radical monoanions to fully reduced ene-1,2-di­thiol­ate dianions. (**d**)–(**e**) Resonance forms within the di­thiol­ene radical monoanion.

Compounds of this type are electrochemically rich and typically support two successive ligand-based one-electron reductions that correspond to the transformations depicted as (**a**) → (**b**) and (**b**) → (**c**) in Fig. 1 ▸. The redox-active mol­ecular orbital has rather modest metal character and is best described as being delocalized among both di­thiol­ene ligands, which individually may be regarded as radical monoanions but which collectively have their spins paired such that the charge-neutral state is diamagnetic. In structure (**c**), both di­thiol­ene ligands are in a fully reduced ene-1,2-di­thiol­ate dianionic state. The potentials at which these reductions occur are quite sensitive to the nature and placement of ring substituents. As part of an effort to more fully map the potential range in which the electron transfers in these complexes occur, we have undertaken the synthesis and characterization of aryl-substituted nickel(II) bis(di­thiol­ene) complexes bearing electron-withdrawing groups. Although a known compound, the nickel(II) bis­(di­thiol­ene) variant with *p*-ClC_6_H_4_– substituents has not been the subject of an X-ray diffraction study, nor has a coordination compound of this ligand with any other metal. We briefly relate here the structural and crystal packing features of [((*p*-ClC_6_H_4_)_2_C_2_S_2_)_2_Ni].

**Figure 2:**
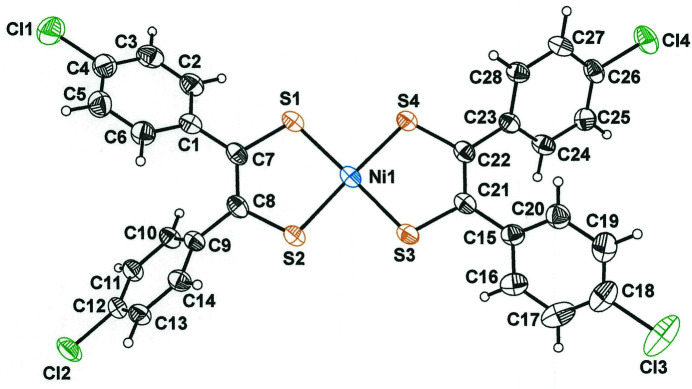
Displacement ellipsoid plot (50% probability level) for bis[1,2-bis­(4-chloro­phen­yl)­ethyl­ene-1,2-dithiol­ato(1−)]nickel(II) with complete atom labeling.

Bis[1,2-bis­(4-chloro­phen­yl)­ethyl­ene-1,2-dithiol­ato(1−)]nickel(II) crystallizes upon a general position in triclinic space group *P* 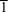 (Fig. 2 ▸). Its idealized point-group symmetry is *D*_2*h*_ if the phenyl groups are either perfectly perpendicular to, or fully planar with, the Ni(S_2_C_2_)_2_ core. However, the four arene rings are canted from the NiS_2_C_2_ chelate ring to which they are attached by values ranging from 38.39 (9)– 53.41 (6)°, the average being 44.87°. A similar description is pertinent to the compounds featuring phenyl, *p*-CH_3_C_6_H_4_–, and *p*-CH_3_OC_6_H_4_– substituents. The averaged S—C bond length in [(*p*-ClC_6_H_4_)_2_C_2_S_2_)_2_Ni] is 1.707 (1) Å. This inter­mediate value between S—C thione (1.63 Å, Rindorf & Carlsen, 1979 ▸; Fu *et al.*, 1997*a* ▸,*b* ▸, 1998 ▸) and vinyl thio­ether (1.74 Å; Tian *et al.*, 1995 ▸; Yu *et al.*, 2011 ▸) bond lengths is due to the presence of some thione character to the bond order in the radical monoanion arising from resonance form (**e**) (Fig. 1 ▸), even as the ligands are coordinating to the metal. Similarly, the C—C_chelate_ bond lengths are between the 1.54 and 1.34 Å values that are typical of carbon–carbon *sp* ^3^–*sp* ^3^ single and *sp* ^2^–*sp* ^2^ double bonds, respectively (Carey & Sundberg, 2000 ▸), further indicating the participation of both resonance forms (**d**) and (**e**) in the electronic structure of bis[1,2-bis­(4-chloro­phen­yl)­ethyl­ene-1,2-dithiol­ato(1−)]nickel(II).

**Figure 3:**
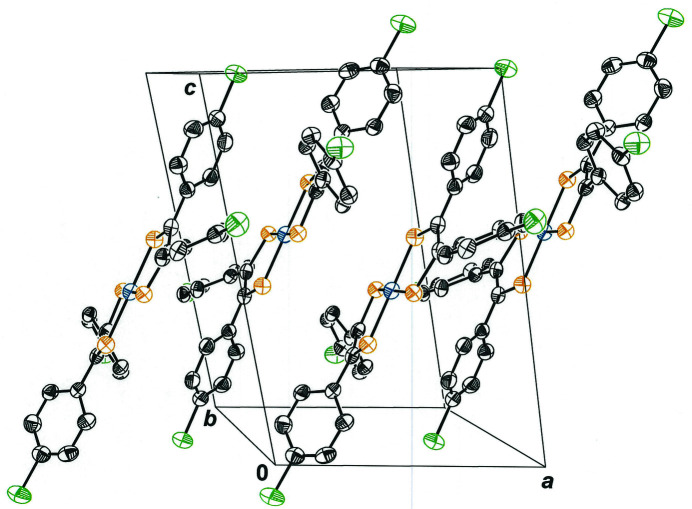
Packing arrangement of mol­ecules of bis[1,2-bis­(4-chloro­phen­yl)­ethyl­ene-1,2-dithiol­ato(1−)]nickel(II) in the unit cell. Ellipsoids are shown at the 50% probability level, and all H atoms are omitted for clarity. Pairs of mol­ecules are related by inversion across the center of symmetry at the center of the *bc* face.

**Figure 4:**
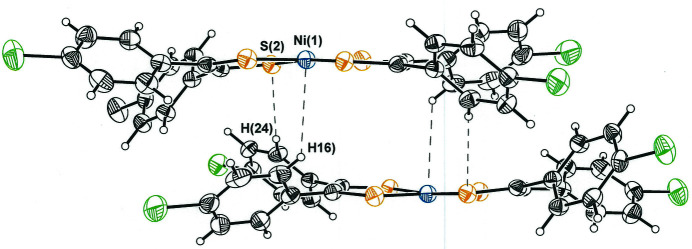
Illustration of the C—H⋯S and C—H⋯Ni contacts that occur between closest pairs of bis[1,2-bis­(4-chloro­phen­yl)­ethyl­ene-1,2-dithiol­ato(1−)]nickel(II) mol­ecules. Ellipsoids are presented at the 50% probability level. Symmetry operation: −*x*, 1 − *y*, 1 − *z*.

**Figure 5:**
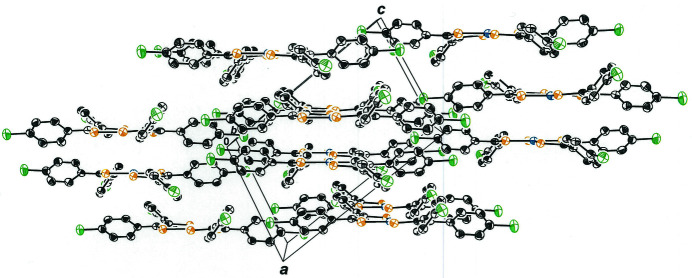
Packing diagram for bis[1,2-bis­(4-chloro­phen­yl)­ethyl­ene-1,2-dithiol­ato(1−)]nickel(II) showing the parallel arrangement of mol­ecules in the direction of the *ac* face diagonal. Displacement ellipsoids are depicted at the 50% probability level.

The packing arrangement for bis[1,2-bis­(4-chloro­phen­yl)­ethyl­ene-1,2-dithiol­ato(1−)]nickel(II) is such that mol­ecules occur in centrosymmetric pairs around the inversion centers that occur at each *bc* face of the cell (Fig. 3 ▸). These pairwise associations juxtapose two mol­ecules in a nearly parallel planar fashion but with an offset that places the phenyl groups of one ligand over the relatively open NiS_4_ inter­ior of its partner mol­ecule. Relatively close inter­molecular C—H⋯S (2.884 Å) and C—H⋯Ni (3.032 Å) contacts are made (Fig. 4 ▸), two each that are related by the inversion symmetry. The C—H⋯S and C—N⋯Ni close contacts are less than the sum of the hydrogen–sulfur and hydrogen–nickel van der Waals radii (Batsanov, 2001 ▸) and appear to be favorable inter­actions that induce a slight but discernible concave bowing of the mol­ecules toward one another (Fig. 4 ▸). This curvature, defined as the angle between the seven-atom mean planes given by each NiS_2_C_2_ chelate and the first carbon atom of each aryl ring, is 11.87 (5)°. It is likely that the angled disposition of some of the aryl substituents with respect to the NiS_2_C_2_ chelate have their origin in these inter­molecular inter­actions. The larger packing arrangement is best described as translations of these centrosymmetric pairs along the *a* axis, the upshot of which is that extended mol­ecular sheets are formed that are oriented in the direction of the *ac* face diagonal (Fig. 5 ▸).

## Synthesis and crystallization

The title compound was prepared from 4,4′-di­chloro­benzil, P_4_S_10_, and NiCl_2_·6H_2_O according to the literature procedure (Schrauzer & Mayweg, 1965 ▸). Yield: 50%. Intense green column-shaped crystals were grown by the diffusion of *tert*-butyl methyl ether vapor into a solution of the title compound in 1,2-dichloro­ethane.

## Refinement

Crystal data, data collection and structure refinement details are summarized in Table 1 ▸. One reflection affected by the beamstop was omitted from the final refinement.

## Supplementary Material

Crystal structure: contains datablock(s) global, I. DOI: 10.1107/S2414314622001481/gg4008sup1.cif

Structure factors: contains datablock(s) I. DOI: 10.1107/S2414314622001481/gg4008Isup2.hkl

CCDC reference: 2150616

Additional supporting information:  crystallographic information; 3D view; checkCIF report

## Figures and Tables

**Table 1:** Experimental details

Crystal data	
Chemical formula	[Ni(C_14_H_8_Cl_2_S_2_)_2_]
*M*_r_	681.16
Crystal system, space group	Triclinic, *P* 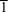
Temperature (K)	170
*a*, *b*, *c* (Å)	9.5487 (4), 11.4141 (4), 15.0254 (6)
α, β, γ (°)	107.486 (2), 94.791 (2), 111.423 (2)
*V* (Å^3^)	1419.16 (10)
*Z*	2
Radiation type	Mo *K*α
μ (mm^−1^)	1.37
Crystal size (mm)	0.27 × 0.15 × 0.10

Data collection	
Diffractometer	Bruker D8 QUEST PHOTON 3 diffractometer
Absorption correction	Numerical (*SADABS*; Krause *et al.*, 2015 ▸)
*T*_min_, *T*_max_	0.76, 0.88
No. of measured, independent and observed [*I* > 2σ(*I*)] reflections	89629, 8009, 5941
*R*_int_	0.056
(sin θ/λ)_max_ (Å^−1^)	0.696

Refinement	
*R*[*F* ^2^ > 2σ(*F* ^2^)], *wR*(*F* ^2^), *S*	0.040, 0.112, 1.03
No. of reflections	8009
No. of parameters	334
H-atom treatment	H-atom parameters constrained
Δρ_max_, Δρ_min_ (e Å^−3^)	0.78, −0.45

Computer programs: *APEX3* and *SAINT* (Bruker, 2020 ▸), *SHELXT* (Sheldrick, 2015*a* ▸), *SHELXL2018/1* (Sheldrick, 2015*b* ▸), *DIAMOND* (Brandenburg & Putz, 2012 ▸), and *SHELXTL* (Sheldrick, 2008 ▸).

## full crystallographic data

### Crystal data

**Table:**

[Ni(C_14_H_8_Cl_2_S_2_)_2_]	*Z* = 2
*M**_r_* = 681.16	*F*(000) = 688
Triclinic, *P*1	*D*_x_ = 1.594 Mg m^−^^3^
*a* = 9.5487 (4) Å	Mo *K*α radiation, λ = 0.71073 Å
*b* = 11.4141 (4) Å	Cell parameters from 9023 reflections
*c* = 15.0254 (6) Å	θ = 2.4–29.5°
α = 107.486 (2)°	µ = 1.37 mm^−^^1^
β = 94.791 (2)°	*T* = 170 K
γ = 111.423 (2)°	Column, intense green
*V* = 1419.16 (10) Å^3^	0.27 × 0.15 × 0.10 mm

### Data collection

**Table:**

Bruker D8 QUEST PHOTON 3 diffractometer	8009 independent reflections
Radiation source: fine-focus sealed tube	5941 reflections with *I* > 2σ(*I*)
Graphite monochromator	*R*_int_ = 0.056
Detector resolution: 7.3910 pixels mm^-1^	θ_max_ = 29.7°, θ_min_ = 2.4°
φ and ω scans	*h* = −13→13
Absorption correction: numerical (*SADABS*; Krause *et al.*, 2015)	*k* = −15→15
*T*_min_ = 0.76, *T*_max_ = 0.88	*l* = −20→20
89629 measured reflections	

### Refinement

**Table:**

Refinement on *F*^2^	Primary atom site location: dual
Least-squares matrix: full	Secondary atom site location: difference Fourier map
*R*[*F*^2^ > 2σ(*F*^2^)] = 0.040	Hydrogen site location: inferred from neighbouring sites
*wR*(*F*^2^) = 0.112	H-atom parameters constrained
*S* = 1.03	*w* = 1/[σ^2^(*F*_o_^2^) + (0.0524*P*)^2^ + 1.0724*P*] where *P* = (*F*_o_^2^ + 2*F*_c_^2^)/3
8009 reflections	(Δ/σ)_max_ = 0.001
334 parameters	Δρ_max_ = 0.78 e Å^−^^3^
0 restraints	Δρ_min_ = −0.45 e Å^−^^3^

### Special details

**Table:**

Experimental. The diffraction data were obtained from sets 11 of frames, each of width 0.5° in ω or φ, collected with scan parameters determined by the "strategy" routine in *APEX3*. The scan time was 15 sec/frame.
Geometry. All esds (except the esd in the dihedral angle between two l.s. planes) are estimated using the full covariance matrix. The cell esds are taken into account individually in the estimation of esds in distances, angles and torsion angles; correlations between esds in cell parameters are only used when they are defined by crystal symmetry. An approximate (isotropic) treatment of cell esds is used for estimating esds involving l.s. planes.
Refinement. Refinement of F^2^ against ALL reflections. The weighted *R*-factor *wR* and goodness of fit S are based on F^2^, conventional *R*-factors *R* are based on F, with F set to zero for negative F^2^. The threshold expression of F^2^ > 2sigma(F^2^) is used only for calculating *R*-factors(gt) *etc*. and is not relevant to the choice of reflections for refinement. *R*-factors based on F^2^ are statistically about twice as large as those based on F, and R– factors based on ALL data will be even larger. H-atoms attached to carbon were placed in calculated positions (C—H = 0.95 Å). All were included as riding contributions with isotropic displacement parameters 1.2 times those of the attached atoms.

### Fractional atomic coordinates and isotropic or equivalent isotropic displacement parameters (Å^2^)

**Table:**

	*x*	*y*	*z*	*U*_iso_*/*U*_eq_	
Ni1	0.35173 (3)	0.63386 (3)	0.56629 (2)	0.03136 (9)	
Cl1	1.01198 (10)	0.89129 (9)	1.11457 (5)	0.0646 (2)	
Cl2	0.44766 (10)	0.05522 (7)	0.82193 (5)	0.05315 (18)	
Cl3	−0.27660 (10)	0.36001 (13)	0.00470 (6)	0.0866 (3)	
Cl4	0.00224 (9)	1.14473 (7)	0.34765 (5)	0.05241 (18)	
S1	0.51018 (7)	0.75530 (5)	0.69986 (4)	0.03387 (13)	
S2	0.35562 (7)	0.45659 (5)	0.58196 (4)	0.03250 (13)	
S3	0.19444 (7)	0.50969 (5)	0.43335 (4)	0.03449 (13)	
S4	0.32801 (6)	0.80720 (5)	0.55486 (4)	0.03159 (12)	
C1	0.6573 (3)	0.7049 (2)	0.83698 (16)	0.0316 (4)	
C2	0.6637 (3)	0.8171 (2)	0.91031 (18)	0.0402 (5)	
H2	0.591392	0.854176	0.902039	0.048*	
C3	0.7730 (3)	0.8754 (3)	0.99479 (19)	0.0458 (6)	
H3	0.776586	0.952416	1.043937	0.055*	
C4	0.8768 (3)	0.8206 (3)	1.00694 (18)	0.0423 (6)	
C5	0.8749 (3)	0.7106 (3)	0.9358 (2)	0.0460 (6)	
H5	0.947798	0.674405	0.944845	0.055*	
C6	0.7664 (3)	0.6533 (3)	0.85115 (18)	0.0392 (5)	
H6	0.765655	0.577825	0.801809	0.047*	
C7	0.5440 (3)	0.6456 (2)	0.74536 (16)	0.0307 (4)	
C8	0.4672 (3)	0.5076 (2)	0.69248 (16)	0.0314 (4)	
C9	0.4672 (2)	0.3986 (2)	0.72654 (16)	0.0296 (4)	
C10	0.4301 (3)	0.3963 (2)	0.81393 (16)	0.0334 (5)	
H10	0.410317	0.468058	0.853727	0.040*	
C11	0.4218 (3)	0.2903 (2)	0.84348 (17)	0.0353 (5)	
H11	0.394413	0.288101	0.902488	0.042*	
C12	0.4541 (3)	0.1880 (2)	0.78565 (17)	0.0343 (5)	
C13	0.4911 (3)	0.1876 (2)	0.69874 (17)	0.0349 (5)	
H13	0.512544	0.116288	0.659864	0.042*	
C14	0.4966 (3)	0.2923 (2)	0.66892 (17)	0.0325 (5)	
H14	0.520569	0.292032	0.608783	0.039*	
C15	0.0353 (3)	0.5525 (2)	0.29789 (17)	0.0330 (5)	
C16	−0.0885 (3)	0.4265 (3)	0.27169 (19)	0.0409 (5)	
H16	−0.106881	0.381348	0.316152	0.049*	
C17	−0.1839 (3)	0.3672 (3)	0.1819 (2)	0.0527 (7)	
H17	−0.268122	0.281852	0.164421	0.063*	
C18	−0.1553 (3)	0.4339 (3)	0.11759 (19)	0.0512 (7)	
C19	−0.0334 (3)	0.5563 (3)	0.14068 (19)	0.0475 (6)	
H19	−0.015078	0.600008	0.095417	0.057*	
C20	0.0625 (3)	0.6152 (3)	0.23024 (18)	0.0395 (5)	
H20	0.147957	0.699344	0.246213	0.047*	
C21	0.1388 (2)	0.6145 (2)	0.39363 (16)	0.0308 (4)	
C22	0.1954 (2)	0.7506 (2)	0.45073 (16)	0.0304 (4)	
C23	0.1474 (2)	0.8490 (2)	0.42788 (15)	0.0295 (4)	
C24	−0.0076 (3)	0.8158 (2)	0.39296 (17)	0.0343 (5)	
H24	−0.082964	0.729787	0.385917	0.041*	
C25	−0.0535 (3)	0.9061 (2)	0.36839 (17)	0.0353 (5)	
H25	−0.159136	0.882127	0.344000	0.042*	
C26	0.0569 (3)	1.0315 (2)	0.37998 (17)	0.0349 (5)	
C27	0.2113 (3)	1.0700 (2)	0.41777 (18)	0.0349 (5)	
H27	0.285423	1.157701	0.427417	0.042*	
C28	0.2555 (3)	0.9781 (2)	0.44116 (17)	0.0319 (4)	
H28	0.360993	1.003321	0.466667	0.038*	

### Atomic displacement parameters (Å^2^)

**Table:**

	*U*^11^	*U*^22^	*U*^33^	*U*^12^	*U*^13^	*U*^23^
Ni1	0.03557 (16)	0.02817 (15)	0.03695 (16)	0.01568 (12)	0.01086 (12)	0.01650 (12)
Cl1	0.0720 (5)	0.0606 (5)	0.0441 (4)	0.0149 (4)	−0.0060 (3)	0.0158 (3)
Cl2	0.0827 (5)	0.0370 (3)	0.0519 (4)	0.0335 (3)	0.0112 (3)	0.0221 (3)
Cl3	0.0569 (5)	0.1278 (9)	0.0417 (4)	0.0310 (5)	−0.0044 (3)	−0.0022 (5)
Cl4	0.0681 (4)	0.0482 (4)	0.0639 (4)	0.0402 (3)	0.0187 (3)	0.0293 (3)
S1	0.0401 (3)	0.0266 (3)	0.0392 (3)	0.0155 (2)	0.0100 (2)	0.0150 (2)
S2	0.0382 (3)	0.0270 (3)	0.0364 (3)	0.0152 (2)	0.0095 (2)	0.0143 (2)
S3	0.0407 (3)	0.0255 (3)	0.0398 (3)	0.0148 (2)	0.0085 (2)	0.0136 (2)
S4	0.0346 (3)	0.0263 (3)	0.0358 (3)	0.0140 (2)	0.0065 (2)	0.0121 (2)
C1	0.0365 (11)	0.0275 (10)	0.0344 (11)	0.0145 (9)	0.0131 (9)	0.0126 (9)
C2	0.0494 (14)	0.0340 (12)	0.0426 (13)	0.0231 (11)	0.0139 (11)	0.0126 (10)
C3	0.0614 (17)	0.0352 (13)	0.0393 (13)	0.0203 (12)	0.0158 (12)	0.0095 (11)
C4	0.0470 (14)	0.0411 (13)	0.0370 (12)	0.0132 (11)	0.0078 (11)	0.0183 (11)
C5	0.0453 (14)	0.0478 (15)	0.0491 (15)	0.0244 (12)	0.0081 (12)	0.0169 (12)
C6	0.0429 (13)	0.0389 (13)	0.0388 (12)	0.0226 (11)	0.0115 (10)	0.0100 (10)
C7	0.0331 (11)	0.0318 (11)	0.0379 (11)	0.0182 (9)	0.0162 (9)	0.0186 (9)
C8	0.0366 (11)	0.0325 (11)	0.0360 (11)	0.0201 (9)	0.0160 (9)	0.0171 (9)
C9	0.0310 (10)	0.0260 (10)	0.0358 (11)	0.0143 (8)	0.0100 (9)	0.0125 (9)
C10	0.0403 (12)	0.0303 (11)	0.0365 (11)	0.0199 (10)	0.0137 (9)	0.0131 (9)
C11	0.0448 (13)	0.0326 (11)	0.0346 (11)	0.0197 (10)	0.0103 (10)	0.0151 (9)
C12	0.0384 (12)	0.0292 (11)	0.0386 (12)	0.0163 (9)	0.0027 (9)	0.0149 (9)
C13	0.0378 (12)	0.0283 (11)	0.0408 (12)	0.0180 (9)	0.0081 (10)	0.0099 (9)
C14	0.0341 (11)	0.0297 (11)	0.0369 (11)	0.0159 (9)	0.0122 (9)	0.0117 (9)
C15	0.0331 (11)	0.0297 (11)	0.0371 (12)	0.0164 (9)	0.0089 (9)	0.0082 (9)
C16	0.0363 (12)	0.0365 (12)	0.0449 (14)	0.0140 (10)	0.0136 (10)	0.0079 (11)
C17	0.0325 (13)	0.0517 (16)	0.0531 (16)	0.0120 (12)	0.0110 (12)	−0.0032 (13)
C18	0.0410 (14)	0.0702 (19)	0.0369 (13)	0.0308 (14)	0.0056 (11)	0.0028 (13)
C19	0.0536 (16)	0.0589 (17)	0.0385 (13)	0.0344 (14)	0.0107 (12)	0.0145 (12)
C20	0.0444 (13)	0.0385 (13)	0.0404 (13)	0.0219 (11)	0.0105 (10)	0.0139 (10)
C21	0.0303 (10)	0.0281 (10)	0.0397 (12)	0.0141 (9)	0.0116 (9)	0.0161 (9)
C22	0.0286 (10)	0.0309 (11)	0.0385 (11)	0.0149 (9)	0.0119 (9)	0.0170 (9)
C23	0.0318 (11)	0.0277 (10)	0.0327 (11)	0.0142 (9)	0.0107 (9)	0.0121 (9)
C24	0.0307 (11)	0.0293 (11)	0.0458 (13)	0.0127 (9)	0.0127 (10)	0.0157 (10)
C25	0.0307 (11)	0.0374 (12)	0.0408 (12)	0.0177 (9)	0.0073 (9)	0.0132 (10)
C26	0.0466 (13)	0.0349 (12)	0.0363 (12)	0.0270 (10)	0.0141 (10)	0.0161 (10)
C27	0.0372 (12)	0.0255 (10)	0.0453 (13)	0.0128 (9)	0.0168 (10)	0.0152 (9)
C28	0.0301 (10)	0.0278 (10)	0.0393 (12)	0.0131 (9)	0.0106 (9)	0.0117 (9)

### Geometric parameters (Å, º)

**Table:**

Ni1—S2	2.1192 (6)	C11—C12	1.383 (3)
Ni1—S3	2.1207 (7)	C11—H11	0.9500
Ni1—S4	2.1261 (6)	C12—C13	1.380 (3)
Ni1—S1	2.1277 (7)	C13—C14	1.382 (3)
Cl1—C4	1.743 (3)	C13—H13	0.9500
Cl2—C12	1.741 (2)	C14—H14	0.9500
Cl3—C18	1.740 (3)	C15—C20	1.400 (3)
Cl4—C26	1.733 (2)	C15—C16	1.400 (3)
S1—C7	1.706 (2)	C15—C21	1.479 (3)
S2—C8	1.704 (2)	C16—C17	1.380 (4)
S3—C21	1.706 (2)	C16—H16	0.9500
S4—C22	1.713 (2)	C17—C18	1.387 (5)
C1—C2	1.393 (3)	C17—H17	0.9500
C1—C6	1.402 (3)	C18—C19	1.371 (4)
C1—C7	1.477 (3)	C19—C20	1.379 (4)
C2—C3	1.381 (4)	C19—H19	0.9500
C2—H2	0.9500	C20—H20	0.9500
C3—C4	1.378 (4)	C21—C22	1.397 (3)
C3—H3	0.9500	C22—C23	1.473 (3)
C4—C5	1.376 (4)	C23—C24	1.397 (3)
C5—C6	1.379 (4)	C23—C28	1.398 (3)
C5—H5	0.9500	C24—C25	1.386 (3)
C6—H6	0.9500	C24—H24	0.9500
C7—C8	1.399 (3)	C25—C26	1.381 (3)
C8—C9	1.480 (3)	C25—H25	0.9500
C9—C10	1.394 (3)	C26—C27	1.388 (3)
C9—C14	1.400 (3)	C27—C28	1.386 (3)
C10—C11	1.387 (3)	C27—H27	0.9500
C10—H10	0.9500	C28—H28	0.9500
			
S2—Ni1—S3	87.80 (2)	C12—C13—H13	120.4
S2—Ni1—S4	174.64 (3)	C14—C13—H13	120.4
S3—Ni1—S4	91.24 (2)	C13—C14—C9	120.6 (2)
S2—Ni1—S1	91.15 (2)	C13—C14—H14	119.7
S3—Ni1—S1	178.94 (2)	C9—C14—H14	119.7
S4—Ni1—S1	89.82 (2)	C20—C15—C16	118.6 (2)
C7—S1—Ni1	105.72 (8)	C20—C15—C21	121.0 (2)
C8—S2—Ni1	105.67 (8)	C16—C15—C21	120.4 (2)
C21—S3—Ni1	105.66 (8)	C17—C16—C15	120.6 (3)
C22—S4—Ni1	105.76 (8)	C17—C16—H16	119.7
C2—C1—C6	117.9 (2)	C15—C16—H16	119.7
C2—C1—C7	121.3 (2)	C16—C17—C18	119.1 (3)
C6—C1—C7	120.7 (2)	C16—C17—H17	120.5
C3—C2—C1	121.3 (2)	C18—C17—H17	120.5
C3—C2—H2	119.3	C19—C18—C17	121.6 (3)
C1—C2—H2	119.3	C19—C18—Cl3	119.3 (2)
C4—C3—C2	119.2 (2)	C17—C18—Cl3	119.1 (2)
C4—C3—H3	120.4	C18—C19—C20	119.3 (3)
C2—C3—H3	120.4	C18—C19—H19	120.3
C5—C4—C3	121.1 (2)	C20—C19—H19	120.3
C5—C4—Cl1	119.5 (2)	C19—C20—C15	120.8 (3)
C3—C4—Cl1	119.4 (2)	C19—C20—H20	119.6
C4—C5—C6	119.6 (2)	C15—C20—H20	119.6
C4—C5—H5	120.2	C22—C21—C15	124.79 (19)
C6—C5—H5	120.2	C22—C21—S3	119.14 (17)
C5—C6—C1	120.9 (2)	C15—C21—S3	116.05 (16)
C5—C6—H6	119.6	C21—C22—C23	124.3 (2)
C1—C6—H6	119.6	C21—C22—S4	118.07 (16)
C8—C7—C1	124.84 (19)	C23—C22—S4	117.60 (17)
C8—C7—S1	118.29 (17)	C24—C23—C28	118.3 (2)
C1—C7—S1	116.84 (16)	C24—C23—C22	120.7 (2)
C7—C8—C9	125.3 (2)	C28—C23—C22	120.95 (19)
C7—C8—S2	118.86 (16)	C25—C24—C23	121.3 (2)
C9—C8—S2	115.76 (17)	C25—C24—H24	119.4
C10—C9—C14	118.9 (2)	C23—C24—H24	119.4
C10—C9—C8	120.74 (19)	C26—C25—C24	118.9 (2)
C14—C9—C8	120.3 (2)	C26—C25—H25	120.6
C11—C10—C9	120.8 (2)	C24—C25—H25	120.6
C11—C10—H10	119.6	C25—C26—C27	121.5 (2)
C9—C10—H10	119.6	C25—C26—Cl4	119.58 (18)
C12—C11—C10	118.9 (2)	C27—C26—Cl4	118.88 (18)
C12—C11—H11	120.5	C28—C27—C26	118.8 (2)
C10—C11—H11	120.5	C28—C27—H27	120.6
C13—C12—C11	121.6 (2)	C26—C27—H27	120.6
C13—C12—Cl2	118.56 (18)	C27—C28—C23	121.1 (2)
C11—C12—Cl2	119.87 (18)	C27—C28—H28	119.4
C12—C13—C14	119.3 (2)	C23—C28—H28	119.4
			
C6—C1—C2—C3	−0.5 (4)	C20—C15—C16—C17	−1.7 (4)
C7—C1—C2—C3	−177.8 (2)	C21—C15—C16—C17	−179.0 (2)
C1—C2—C3—C4	−0.6 (4)	C15—C16—C17—C18	0.3 (4)
C2—C3—C4—C5	1.2 (4)	C16—C17—C18—C19	0.9 (4)
C2—C3—C4—Cl1	−178.4 (2)	C16—C17—C18—Cl3	−179.4 (2)
C3—C4—C5—C6	−0.6 (4)	C17—C18—C19—C20	−0.6 (4)
Cl1—C4—C5—C6	179.0 (2)	Cl3—C18—C19—C20	179.73 (19)
C4—C5—C6—C1	−0.5 (4)	C18—C19—C20—C15	−0.9 (4)
C2—C1—C6—C5	1.1 (4)	C16—C15—C20—C19	2.0 (3)
C7—C1—C6—C5	178.4 (2)	C21—C15—C20—C19	179.3 (2)
C2—C1—C7—C8	−142.3 (2)	C20—C15—C21—C22	44.4 (3)
C6—C1—C7—C8	40.5 (3)	C16—C15—C21—C22	−138.4 (2)
C2—C1—C7—S1	39.8 (3)	C20—C15—C21—S3	−133.8 (2)
C6—C1—C7—S1	−137.4 (2)	C16—C15—C21—S3	43.5 (3)
Ni1—S1—C7—C8	−0.78 (19)	Ni1—S3—C21—C22	−2.11 (19)
Ni1—S1—C7—C1	177.20 (14)	Ni1—S3—C21—C15	176.18 (15)
C1—C7—C8—C9	10.5 (3)	C15—C21—C22—C23	6.6 (3)
S1—C7—C8—C9	−171.64 (17)	S3—C21—C22—C23	−175.30 (17)
C1—C7—C8—S2	−172.99 (17)	C15—C21—C22—S4	−174.10 (17)
S1—C7—C8—S2	4.8 (3)	S3—C21—C22—S4	4.0 (3)
Ni1—S2—C8—C7	−6.26 (19)	Ni1—S4—C22—C21	−3.77 (19)
Ni1—S2—C8—C9	170.53 (14)	Ni1—S4—C22—C23	175.60 (14)
C7—C8—C9—C10	51.3 (3)	C21—C22—C23—C24	42.9 (3)
S2—C8—C9—C10	−125.3 (2)	S4—C22—C23—C24	−136.43 (19)
C7—C8—C9—C14	−132.1 (2)	C21—C22—C23—C28	−137.6 (2)
S2—C8—C9—C14	51.3 (3)	S4—C22—C23—C28	43.1 (3)
C14—C9—C10—C11	−0.2 (3)	C28—C23—C24—C25	2.5 (3)
C8—C9—C10—C11	176.4 (2)	C22—C23—C24—C25	−178.0 (2)
C9—C10—C11—C12	1.3 (4)	C23—C24—C25—C26	−0.7 (4)
C10—C11—C12—C13	−1.3 (4)	C24—C25—C26—C27	−1.7 (4)
C10—C11—C12—Cl2	178.92 (18)	C24—C25—C26—Cl4	178.84 (18)
C11—C12—C13—C14	0.2 (4)	C25—C26—C27—C28	2.2 (4)
Cl2—C12—C13—C14	179.98 (18)	Cl4—C26—C27—C28	−178.26 (18)
C12—C13—C14—C9	0.9 (3)	C26—C27—C28—C23	−0.4 (3)
C10—C9—C14—C13	−0.9 (3)	C24—C23—C28—C27	−1.9 (3)
C8—C9—C14—C13	−177.6 (2)	C22—C23—C28—C27	178.5 (2)
